# IRB practices and policies regarding the secondary research use of biospecimens

**DOI:** 10.1186/s12910-015-0020-1

**Published:** 2015-05-08

**Authors:** Aaron J Goldenberg, Karen J Maschke, Steven Joffe, Jeffrey R Botkin, Erin Rothwell, Thomas H Murray, Rebecca Anderson, Nicole Deming, Beth F Rosenthal, Suzanne M Rivera

**Affiliations:** Department of Bioethics, TA212 School of Medicine, Case Western Reserve University, 10900 Euclid Ave., Cleveland, OH 44106 USA; The Hastings Center and the Centre for Biomedical Ethics, Garrison, New York USA; Department of Medical Ethics and Health Policy, University of Pennsylvania Perelman School of Medicine, Philadelphia, Pennsylvania USA; Department of Pediatrics, University of Utah, Salt Lake City, Utah USA; Yong Loo Lin School of Medicine, National University of Singapore, Singapore, Singapore

**Keywords:** IRBs, Policy, Biobanks, Biospecimens, Data sharing, Genetic research

## Abstract

**Background:**

As sharing and secondary research use of biospecimens increases, IRBs and researchers face the challenge of protecting and respecting donors without comprehensive regulations addressing the human subject protection issues posed by biobanking. Variation in IRB biobanking policies about these issues has not been well documented.

**Methods:**

This paper reports on data from a survey of IRB Administrative Directors from 60 institutions affiliated with the Clinical and Translation Science Awards (CTSAs) about their policies and practices regarding secondary use and sharing of biospecimens. Specifically, IRB ADs were asked about consent for future use of biospecimens, assignment of risk for studies using biobanked specimens, and sharing of biospecimens/data.

**Results:**

Our data indicate that IRBs take varying approaches to protocol review, risk assessment, and data sharing, especially when specimens are not anonymized.

**Conclusion:**

Unclear or divergent policies regarding biospecimen research among IRBs may constitute a barrier to advancing genetic studies and to inter-institutional collaboration, given different institutional requirements for human subjects protections.

**Electronic supplementary material:**

The online version of this article (doi:10.1186/s12910-015-0020-1) contains supplementary material, which is available to authorized users.

## Background

In recent years, questions about how to apply human subjects protections to biobanking-related research have challenged institutional review boards (IRBs) [1-4]. These include: what consent approaches are appropriate for collecting, storing, and using research participants’ biospecimens and their associated data; whether and how language in original consent forms should be considered when conducting secondary studies using stored biospecimens; what level of risk (no more than minimal or greater than minimal) should be assigned to studies using stored biospecimens; and what requirements should be imposed for sharing biospecimens with internal and external researchers [4-7]? Regulations governing research involving human subjects do not explicitly address the unique human subjects protections challenges presented by research with human biospecimens, whether the specimens in question are collected prospectively or previously stored. Furthermore, inconsistent guidance from government agencies, research organizations, and professional societies may make it difficult for researchers and IRBs to determine how biospecimens/data should be collected, stored, and used in ways that protect the rights and welfare of biospecimen donors while advancing genetic and other biomedical research [6].

The challenges IRBs face with regard to human subjects protections for biobanking-related research may be magnified when researchers at one institution seek to share biospecimens with external researchers or to conduct research with biospecimens/data they obtain from other institutions [8]. Variations across IRBs in policies and procedures regarding human subjects protections for biobanking-related research add to the complexity of cross-institutional collaboration and may hinder certain kinds of potentially beneficial research endeavors [9]. Little is known about the degree of variety among IRB policies and practices regarding biobanking-related research, particularly genetic research and research involving inter-institutional collaboration or data sharing [7]. The present survey of IRB Administrative Directors (ADs) represents one aim of a larger NIH-funded study designed to better understand the range and variation of IRB policies and practices regarding human subject protections in the context of the collection, storage and use of biospecimens and associated data.

The purposes of the larger study are to 1) describe the usual practices and attitudes of stakeholder groups at Clinical and Translational Science Award (CTSA) institutions (including IRB administrators) about specific policy options in order to address and understand potential barriers to collaborative research; 2) understand how institutional policies controlling the creation and use of biobanks are developed; and 3) analyze, through an interdisciplinary process, the ethical and regulatory issues that frame policies on informed consent and sharing biospecimens/data across institutions, and 4) develop a set of policy/practice recommendations.

Funded by the National Center for Advancing Translational Sciences, a part of the National Institutes of Health (NIH), the CTSA program represents a core infrastructure for publicly funded translational research in the United States [10]. It is a key example of the growing trend toward promoting collaborative research within and across sites in order to facilitate interventions from the laboratory bench to the bedside. This national consortium exemplifies the growing importance of collaborative research, in which member institutions are expected to transform clinical and translational research and cooperate within and across sites. [10] In this report, we describe key human subjects protection issues that IRBs at CTSA institutions confront as they review biobanking-related research protocols.

## Methods

To characterize IRB practices, we surveyed IRB Administrative Directors (ADs) from every CTSA program established as of 2012 (N = 60). These are the individuals who supervise and oversee IRB operations, are most likely responsible for promulgating IRB policies and procedures, and typically are most knowledgeable about usual IRB practices at their institutions. We recruited IRB ADs from all 60 CTSA sites (as of 2012 when surveys were conducted) as the target sample. For CTSAs consisting of multiple institutions represented by numerous IRBs, we surveyed the AD of the IRB at the institution within that CTSA which received the most NIH funding (according to the NIH Research Portfolio Online Reporting Tool (RePORT) [11].

### Survey design

We sought to learn how research institutions in the CTSA consortium approach the protection of research participants in biobanking-related research. We focused on institutions’ IRB policies and usual practices for obtaining consent for research involving biobanking, and on sharing biospecimens/data across academic institutions. Based on the expertise of the research team and a review of the literature, we developed a preliminary 33-item survey that we pretested with 6 local IRB representatives to assess ease of survey administration and face validity. We then developed a final survey comprising 37 questions, primarily using a five-point Likert answer scale (Additional file 1).

Throughout the survey we used the term “Biospecimens and Data” to reflect that in the context of specimen research, the samples themselves are valuable insofar as they are associated with or help to generate data. Through this survey, we wanted to capture Administrative Directors’ views on practices and policies surrounding these as one concept, to better reflect the context of specimen research. We will therefore use the term “biospecimen” or “specimen” throughout the rest of this paper to represent both the sample itself, data associated or linked with that sample, and or data generated from using a sample.

### Survey sample and administration of survey

After identifying eligible IRB ADs at each CTSA institution in our target sample (using institutional websites and personal contacts at some of the institutions), a member of the research team called each potential respondent to verify his/her position at the institution and to confirm all contact information. Study staff followed up with a telephone call to explain the project, determine the AD’s interest in participating in the survey, and schedule a time to administer the survey via phone. We sent an email to confirm the survey session time, and attached a copy of the survey and an information sheet about the study in advance of the scheduled survey.

Surveys were administered by telephone, and took place between June and August of 2012. Verbal consent was obtained from each participant prior to beginning data collection. On average, surveys lasted approximately 45 minutes. In addition to the investigator administering the survey, two additional project staff members were present during the phone calls in order to ensure accurate capture of participants’ responses. This project was reviewed and approved by the Institutional Review Board of University Hospitals in Cleveland, OH. The IRB waived the requirement for documentation of informed consent for participation in this survey.

### Data management and analysis

Survey data were managed using Research Electronic Data Capture Application (REDCap) software, hosted at Case Western Reserve University (CWRU). REDCap is a secure, web-based application designed to support data capture and analysis for research studies [12]. Survey data were de-identified and entered into the REDCap database. All descriptive analyses were conducted using REDCap software.

## Results

We surveyed 51 IRB ADs, representing an 85% response rate. Respondents’ length of experience in their positions ranged from less than 1 year to 22 years (median 8.8 years). Most (57%) of the IRBs represented by the ADs reviewed more than 100 new protocols involving genetic research each year. Seventy-five percent of the institutions’ IRBs had received the optional IRB accreditation by the Association for the Accreditation of Human Research Protection Programs (AAHRPP). The following results sections focus on two primary areas of inquiry: 1) general IRB practices regarding risk and review requirements for studies using previously stored biospecimens (and associated data) within their institutions, and 2) IRB practices regarding the sharing of biospecimens with researchers at other (“outside”) institutions.

### IRB practices regarding risk and review requirements for studies using stored biospecimens

#### Level of risk attributed to research using biobanked specimens/data

We asked respondents, “What level of risk [their] IRBs would typically assign a study using stored specimens and data?” Respondents were then asked to rate the level of risk their IRBs would typically assign to a proposed study using stored specimens with differing degrees of identifiers (Table 1). We defined *anonymized biospecimens* as having no identifiers or codes linked to identifying information about the donors and *coded/de-identified biospecimens* as being linked to identifying information about the donors, without the researcher having access to the key that links the code to identifying information.

**Table 1:** **Level of risk associated with the use of stored biospecimens**

	**No greater than minimal risk n (%)**	**Greater than minimal risk n (%)**	**Unsure/don’t know n (%)**
Anonymized	50 (98)	0 (0)	1 (2)
Coded	44 (86)	5 (10)	2 (4)
Identified	23 (45)	16 (31)	12 (24)

For anonymized and de-identified specimens, there was general agreement across institutions about the assignment of risk. Ninety-eight percent of respondents said their IRBs consider studies using anonymized biospecimens to be no greater than minimal risk. Similarly, 86% of respondents said that studies using coded specimens also would be considered no greater than minimal risk. However, when attributing risk to the proposed use of identified specimens and data, responses were more heterogeneous. While 45% of respondents believed their IRBs would classify these kinds of studies as “no greater than minimal risk,” 31% felt their IRBs would assign the research as “greater than minimal risk,” and 23% of IRB ADs were either unsure what risk their IRBs would assign or felt that it would depend on the study and specific details regarding the proposed research.

#### Practices regarding review of protocols using biobanked biospecimens

We then asked the ADs “whether [their] IRBs would typically require a researcher to submit information about their study (using biospecimens) to determine if the study is exempt from the oversight requirements of the Common Rule or require further IRB review.” While there was some consistency in answers across ADs, their IRBs’ approaches depended on the identifiability of the samples in question. For anonymized data, a majority (61%) of ADs said their IRBs would usually or always require a researcher to submit his or her study for an initial review, while 39% said that their IRBs would never, rarely, or sometimes require this practice. When specimens are coded/de-identified, 82% of ADs said their IRBs would usually or always require researchers to submit study information, and 100% of ADs reported that when studies utilize identifiable specimens their IRB would require the researchers to submit information to the IRB about their studies.

#### IRB practices for reviewing original consent language

Respondents also were asked if their IRBs would “typically review the original consent language” from the studies for which participants’ data and specimens were collected, in order to assess whether a new use would fall within the scope of the original consent form. Thirty-four percent of respondents reported that their IRBs would review the original consent form language if the new study proposed to use only anonymized specimens. This number increased to 61% for coded/de-identified specimens and to 84% for identifiable specimens.

There also appear to be differences in what IRBs look for when reviewing a consent form to determine whether a proposed new study involving banked specimens falls within the scope of the uses described in the original consent form authorizing their collection or storage for research. Thirty percent of respondents said their IRBs would want to determine whether the new study is “consistent” with the original uses described in the consent form, whereas 69% said their IRBs would want to know that the study is “not inconsistent” with uses described. This distinction may result in material differences for researchers. The approach of seeking consistency requires congruence, which is more limiting. The approach of verifying a lack of inconsistency can result in a broader scope of use than the original approving IRB and research participants may have imagined.

#### Acceptability of research practices for studies that fall outside the original scope of consent

Respondents were asked about “the acceptability of different approaches to resolve issues related to stored sample use, when a proposed study is determined to fall outside the original scope of consent” (Table 2). A majority (78%) of ADs reported that their IRBs would either encourage or require seeking new consent from biospecimen donors/research participants. However, there were more diverse responses with respect to the option to anonymize the data at the time of the new study. Thirty-one percent of IRBs prohibit this option, while 14% discourage it, 24% permit it, 17% encourage it and 14% require it. Nearly 37% of IRBs prohibit or discourage a request for a waiver of informed consent whereas almost 24% encourage or require this option when faced with a request for a new use outside the scope of the original consent used for specimen collection.

**Table 2:** **Acceptability of data management options for proposed studies that fall outside the scope of an original consent**

	**Prohibit n (%)**	**Discourage n (%)**	**Permit n (%)**	**Encourage n (%)**	**Require n (%)**
Anonymize the data	16 (31)	7 (14)	12 (24)	9 (18)	7 (14)
Seek new consent from participants	0 (0)	0 (0)	21.6 (11)	23 (45)	17 (33)
Consider an application for waiver	5 (10)	14 (28)	20 (39)	8 (16)	4 (8)

### Biospecimen sharing with “outside” institutions

With regard to sharing biospecimens/data with researchers outside of their own institutions, we asked the ADs to, “imagine that a researcher at your institution has a collection of biospecimens/data that he would like to share with a researcher outside your institution.” Respondents were then asked whether their institutions’ IRBs would require either 1) submission of information about the proposed study and/or 2) documentation of the outside institution’s IRB authorization for the proposed study. Responses varied depending on the identifiability of the biospecimens and whether the original investigator who collected the specimens would be involved in the proposed study.

#### IRB requirements for biospecimen/data sharing without original researcher involvement (non-collaborative proposal)

When a researcher wishes only to *provide* biospecimens to another researcher, and will not be involved in the new study, 29% of IRBs would require any information about the proposed study and 24% would require documentation from the external (receiving) researcher’s IRB when the biospecimens are *anonymized* (Table 3). When biospecimens are *coded/de-identified,* 43% of IRBs typically would require an information review and 65% would require documentation of the outside institution’s IRB authorization. If the specimens were *identified*, approximately 58% of IRBs would require both information about the proposed study and evidence of IRB authorization at the receiving investigator’s institution.

**Table 3:** **IRB requirements when the original researcher will or will NOT be involved in proposed study using previously collected biospecimens**

		**Original researcher NOT involved**	**Original researcher involved**
**Level of Identifiability**		**Yes/Usually n (%)**	**Yes/Usually n (%)**
Anonymized	Require submission of information about the new study	15 (29)	40 (78)
	Require submission of documentation of review from external researcher’s IRB	12 (24)	28 (55)
Coded	Require submission of information about the new study	22 (43)	48 (94)
	Require submission of documentation of review from external researcher’s IRB	18 (35)	36 (71)
Identified*	Require submission of information about the new study	30 (59)	51 (100)
	Require submission of documentation of review from external researcher’s IRB	30 (59)	40 (75)

*Data missing from 2 respondents.

#### IRB requirements for biospecimen sharing with original researcher involvement (collaborative proposal)

When the researcher who collected the biospecimens will collaborate with researchers at another institution to conduct a new study using the existing biospecimens, even when those specimens have been anonymized, 55% of the IRBs would require documentation of IRB authorization by the collaborators’ external site(s) and 78% would require submission of information about the new study (Table 3). When the biospecimens are coded/de-identified, 94% would require information about the study and 71% would want to receive the outside institution’s IRB authorization. Lastly, when biospecimens are identifiable, 100% of institutions would require study information and 75% would require IRB documentation.

#### IRB requirements for proposed projects using biospecimens from outside institutions

Respondents were asked to, “Imagine that a researcher at your institution proposes a study that would involve only biospecimens that she would obtain from a source outside your institution.” Respondents were then asked whether their IRBs would require a review of the original consent form before a researcher at their institutions could use biospecimens provided by another institution. Thirty-nine percent said their IRBs always or usually review the original consent form when biospecimens are coded/de-identified, whereas 65% said their IRBs would always require review when biospecimens are identifiable.

## Discussion

To obtain biospecimens needed for genetic and genomic studies, researchers may utilize collections within their own institutions, acquire samples from biobanks at other institutions, or work collaboratively with researchers at other institutions who may have larger or more diverse collections than their own [9,13,14]. However, little attention has been paid to how IRBs across major biomedical research institutions approach the review of studies that use stored biospecimens, or their policies and procedures regarding inter-institutional sharing of biospecimens. Our results shed light on these questions from the perspective of IRB Administrative Directors at institutions associated with CTSAs.

First, much of the variability in ADs’ responses regarding decisions about when to review studies that utilize stored biospecimens was related to the level of identifiability of those biospecimens. Generally, the more identifiable biospecimens are, the more likely IRBs are to require review of both a new protocol and the original consent form used when the biospecimens were collected from donors. All IRBs reported requiring an initial review of studies using identifiable samples. Conversely, there was more variability in responses for anonymized biospecimens. ADs reported reviewing studies less frequently when the specimens used in a new protocol were anonymized. Although this is not surprising given that anonymized specimens are not currently categorized as human subjects research according to U.S. regulations, most ADs (61%) in this study reported that an initial review by their IRBs would be necessary even when specimens are anonymized. This may be due to the fact that many IRBs expect to make the determination whether protocols involve human subjects research. However, a significant minority of ADs surveyed said their IRBs do not require review for these kinds of studies.

While there was more uniformity around decisions to review studies using identifiable biospecimens, there was a high degree of variation across respondents when assessing the risk levels of those studies. About half of the ADs surveyed said their IRBs would categorize research using identifiable samples as “no more than minimal risk.” Alternatively, one third of ADs responded that their IRBs would typically categorize research using identifiable specimens as “greater than minimal risk.” Further, a number of ADs were either unsure what level of risk their IRBs would assign a study using identifiable specimens, or thought their IRB would assess risk on a case-by-case basis. The actual reasons for this variation are unknown given that ADs were not presented with scenarios depicting specific research projects that might raise different levels of risks. We therefore do not know what types of studies or risks ADs had in mind when responding. Nevertheless, these data do provide us with an important baseline to compare how identifiability may influence an IRB’s categorization of risk for studies proposing to use stored biospecimens.

Unclear policies or inconsistent attitudes regarding risk assessment may hinder a researcher’s ability to anticipate and address the human subject protections needed when designing a research protocol. In addition, it may be more difficult for investigators to collaborate across institutions with divergent policies or practices regarding review of protocols, or regarding the assessment of risks posed by studies that utilize biospecimens, if those policies create barriers to developing a cohesive protocol across sites. It could be challenging, for example, to develop a multi-site collaboration if each institution’s IRB has different expectations regarding the need for obtaining new consent from donors about the new proposed use(s).

Second, it appears that IRBs differ in their approaches to data management when a new protocol is deemed to fall outside the scope of the original consent form used when biospecimens were collected. While all ADs indicated that their IRBs would either allow or encourage seeking new consent from donors, there was greater variability with regard to the approach of post-hoc anonymization of specimens and the applicability of a waiver of informed consent. This variation could present a barrier to collaboration if, for example, an IRB at one project site would allow specimens to be de-identified and re-used without obtaining new consent from the donors while another would require donors to give consent for each new use. It should be noted here that the anonymization and re-use, without new consent, of existing specimens is a matter of some controversy among bioethicists concerned specifically with human research protections. While some argue persuasively that biospecimen anonymization reduces risks to levels so low that such studies would not even qualify for regulatory protection under the current regulatory framework (and that re-use of specimens is a way to maximize the principle of beneficence), others raise legitimate concerns about whether such a practice may violate the expectations of the original donors and thereby create a dignitary harm [2,6,15].

Lastly, we found variation in IRB practices regarding the sharing of biospecimens with researchers at outside institutions. When researchers wish merely to share biospecimens across institutions but not participate in the new uses of those specimens once they are shared, most ADs indicated that their IRBs would not need to review information regarding the study proposals or IRB approvals from the outside institutions when biospecimens are anonymized or coded. However, if a researcher wanted to share identified biospecimens, we saw a higher level of variation across IRBs. A slight majority would want information regarding the proposal and documentation of review from the external researcher’s IRB.

By contrast, when biospecimen sharing included participation of the original researchers who collected the specimens in the new research project, most ADs reported their IRBs’ expectations to receive information about the protocol and to review documentation from the external researcher’s IRB. When the proposed study involves collaboration across institutions, and proposes to use anonymized or coded specimens, about half of ADs also indicated a need to review protocol materials. While this variation may reflect differing practices regarding the review of studies using coded or anonymized specimens more generally, our findings indicate that IRBs distinguish between simple biospecimen sharing and actual collaborations between institutions that involve the uses of stored specimens. If this is the case, differing policies regarding the need to review studies involving anonymized or coded specimens may be a challenge for collaborating researchers. For example, if one institution requires review of an outside collaborator’s protocol, but that collaborating institution does not require review for protocols using anonymized samples, researchers may find themselves in a “Catch-22” scenario.

All of these issues may become increasingly problematic if the U.S. Department of Health and Human Services (DHHS) issues a regulation that would require informed consent from donors (or biospecimen contributors) for any future uses of all specimens. Released in 2009, the Advance Notice of Proposed Rule Making (ANPRM) proposes a brief, general consent for any future uses of biospecimens, even if they are stripped of identifiers [16-18]. Because of these proposed changes, IRBs may start seeing more protocols proposing to use stored biospecimens in anticipation of potential policy changes. Even if the DHHS does not issue a new rule requiring consent for the use of all specimens (including those that are anonymized), an ongoing discussion about the identifiability of DNA among geneticists, ethicists and others has included calls for more consistent federal policies regarding identifiability of DNA and human subject protections [19,20]. Our data further highlight the need for national guidelines from bodies like the Office for Human Research Protections (OHRP) that would promote clear and interoperable IRB practices regarding use of banked specimens.

It is also crucial to recognize that IRB differences in approaches to biobanking research may reflect normative disagreements among researchers, participants, IRB members, and bioethics scholars regarding the interpretation of federal policies that govern human subjects research. Therefore, while variation in the policies and practices of IRBs may be seen as a barrier to biospecimens sharing or collaborative research, it also could reflect meaningful normative differences about human subjects protections supported by the inherent flexibility in the regulations. It will be increasingly important to consider how to balance the need to support IRBs’ ability to interpret policies within their own institutional contexts while reducing barriers to inter-institutional research. While interoperability may not always be warranted or even possible, a set of guiding principles to address these concerns would nonetheless be valuable.

## Limitations

This study has a number of limitations. First, our sample consisted of IRB Administrative Directors from larger research institutions associated with CTSAs, and may not represent the practices of all IRBs, including institutions with less research intensity or fewer resources. More research with non-CTSA institutions would help to better understand the practices of a wider range of IRBs. Second, the survey itself did not capture qualitative responses from ADs, limiting our insight into why particular approaches are taken by their IRB or how those practices evolved. We did, however, conduct a subsequent series of in-depth interviews with a number of the ADs from our sample [21].

## Conclusion

Increasingly, translational genomic research requires large numbers of biospecimens from diverse populations and environments. Because this need for larger quantities of biospecimens often exceeds the capacity of a single institution, there is an increasing interest in the collection, storage, and sharing of human biospecimens and associated data across institutions. Our data indicate that when reviewing proposed studies that utilize biospecimens, IRBs at major academic health centers may take differing approaches to protocol review, risk assessment, and data sharing. These differing practices may result from IRBs’ local interpretations of federal guidelines or institutional practice, and are not inherently problematic for the conduct of research. IRBs should be able to set requirements that are consistent with federal standards but also reflect institutional needs and legitimate differences in interpretation of legal requirements and ethical norms. However, as the uses of stored biospecimens for research increase, a lack of interoperable policies across institutions may present barriers to inter-institutional sharing of biospecimens. Divergent or unclear approaches to the review of studies utilizing biospecimens may make navigating the IRB process more difficult or potentially discourage collaboration between researchers across institutions. More data are needed to determine whether differing IRB policies are in fact making collaborative genetic research more difficult for researchers. Our data do, however, indicate that reducing variability in IRB approaches could improve the ability to conduct genetic research using biobanked biospecimens within and across academic health centers.

## Additional file

Additional file 1:
**Biospecimen Research Questionnaire.**
