# Supplementary material for: IRB practices and policies regarding the secondary research use of biospecimens
Source: BMC Med Ethics. 2015 May 8;16:32. doi: 10.1186/s12910-015-0020-1 (PMC4426182; doi:10.1186/s12910-015-0020-1)
Supplement: Additional file 1: — Biospecimen Research Questionnaire. [file 12910_2015_20_MOESM1_ESM.pdf]

Institution and Respondent(s):
Institution ID #:
Date:

IRB Policies and Practices for the
Collection, Storage, and Research Use of Human Biospecimens and Associated Data for Genetic Research

Welcome! We are writing to you as the IRB Administrative Director of a major academic institution.

The questions focus mainly on your IRB's policies and usual practices with regard to obtaining consent for genetic research involving the collection, storage, and use of biospecimens and associated data, and on sharing biospecimens/data across academic institutions for genetic research. The purpose of this survey is to gather your professional opinion as an IRB leader about how research institutions approach the protection of research participants in collaborative genetic research. This survey is part of a larger project supported by a grant (#1R01 4G005691-01A1) from the National Human Genome Research Institute at NIH.

Before you begin, please note:

- Please base your responses on your experience regarding policies and usual practices at your institution.
- A review of your records is not needed; please just answer to the best of your ability. There are no right or wrong answers. It is fine if you would like to get input from your IRB chairs and staff.
- Completing this survey will take about 30 minutes of your time. The questions are primarily multiple choice, with a few open ended items.
- Taking part in this survey is completely voluntary. With the exception of Questions #1 and #2, which confirm your eligibility for this survey, you may skip any question that you prefer not to answer.

Your responses will be kept completely confidential and results will be reported in aggregate only. If you have any questions or concerns about your rights as a research participant, please contact the IRB Office at University Hospitals Case Medical Center (serving as the IRB of record for Case Western Reserve University) at 216-844-1529. If you would like a copy of the aggregate results, please check the box at the end of the survey.

We very much appreciate your time and assistance, and look forward to receiving your input on these important topics. If you have any questions about this project, please do not hesitate to contact the Principal Investigator, Dr. Leona Cuttler, at Leona.cuttler@case.edu or 216-844-6253 or the Project Manager, Ann Nevar, at ann.nevar@uhhospitals.org or 216-844-6253.

Institution and Respondent(s):
Institution ID #:
Date:

DEFINITIONS

We use the terms below in several sections of the survey. Please return to this page as needed to review the definitions. at any time.

- "Your IRB" means the system of IRBs that you oversee (i.e., if you are at an institution that has multiple IRBs)
- "Unspecified future use" means the purpose/focus of the future research is stated broadly, allowing for the possibility of a range of studies rather than restricting them to a particular medical condition or pre-defined research questions
- "Anonymized" means the researcher will use samples/data that have no identifiers or codes linked to identifying information
- "Coded" means the researcher will use samples/data that have a code that is linked to identifying information, but the researcher has no access to the key that links the code to identifying information
- "Identified" means the researcher will use samples/data that have personal identifiers, or a code for which the researcher has access to the key that links the code to identifying information
- "Tiered consent" means that, in the written consent, research participants are offered several choices regarding aspects of future research such as what types of research can be conducted with their biospecimens and whether they can be recontacted

Institution and Respondent(s):
Institution ID #:
Date:

I. YOU AND YOUR WORK

1. For how many years have you been an IRB Administrative Director? If your service has been intermittent, please count the total number of years.

    _____ years

2. Approximately how many new protocols **involving human genetic research** did your IRB review in the last year (2011)?

    _____ None
    _____ 1 - 20
    _____ 21 - 100
    _____ More than 100
    _____ I am unable to estimate even an approximate number

3. Approximately how many new protocols **overall** (i.e., genetic and non-genetic) did your IRB review in the last year (2011)?

    _____ 1 - 100
    _____ 101 - 500
    _____ 501-1000
    _____ >1000

Institution and Respondent(s):
Institution ID #:
Date:

II. CONSENTS FOR THE COLLECTION AND STORAGE OF BIOSPECIMENS AND ASSOCIATED DATA FOR GENETIC RESEARCH

Case: For Questions 4 – 10, imagine that a researcher at your institution plans to collect biospecimens and associated data for a specific study, but also wants to store them for future research.

What approach would your IRB *typically take* regarding the acceptability of the following potential aspects of such a protocol:

In the situation described above, what approach would your IRB typically take with regard to….

4. …Asking participants' consent to store biospecimens/data for **future research use?** Our IRB would typically**:**

○ Prohibit this	○ Discourage this	○ Permit, but neither encourage/discourage	○ Encourage this	○ Require this

5. …Asking participants' consent for **ongoing access to their medical records** (to obtain longitudinal data with which to annotate biospecimens)? Our IRB would typically:

○ Prohibit this	○ Discourage this	○ Permit, but neither encourage/discourage	○ Encourage this	○ Require this

6. …Offering participants a **choice about storage for future research use** (i.e., allowing individuals to participate in the immediate study without necessarily agreeing to anything more)? Our IRB would typically:

○ Prohibit this	○ Discourage this	○ Permit, but neither encourage/discourage	○ Encourage this	○ Require this

Institution and Respondent(s):
Institution ID #:
Date:

7. …Offering participants **choices about how their biospecimens/data are used** in future research (sometimes called "tiered consent")? Our IRB would typically:

○	○	○	○	○
Prohibit this	Discourage this	Permit, but neither encourage/discourage	Encourage this	Require this

If your IRB prohibits or has never approved a tiered consent form, skip to Question 9.

8. …Offering participants each of the following **kinds of choices in a tiered consent form**:

	Prohibit this	Discourage this	Permit, but neither encourage/ discourage	Encourage this	Require this
a. The option to restrict the types of *medical conditions* that could be studied (e.g., breast cancer rather than any type of cancer)	○	○	○	○	○
b. The option to restrict the types of *researchers* who could use the biospecimens/data (e.g., academic, industry, or government)	○	○	○	○	○
c. The option to be contacted to request *specific consent* for future uses of biospecimens/data (e.g., consent to specific study of diabetes with option to be contacted for future use of biospecimens/data)	○	○	○	○	○
d. The option to prohibit submission of data to *centralized repositories* (e.g., dbGaP)	○	○	○	○	○

Institution and Respondent(s):
Institution ID #:
Date:

9. …Offering participants each of the following **other options** in the consent form:

	Prohibit this	Discourage this	Permit, but neither encourage/ discourage	Encourage this	Require this
a. To allow (or not) recontact about *participation in other studies* that require separate consent	○	○	○	○	○
b. To be contacted (or not) about *individual research results*	○	○	○	○	○
c. To receive (or not) *aggregate research results*(i.e., overall findings from studies done using stored biospecimens/data)	○	○	○	○	○

10. Please share any thoughts that might help clarify your IRB's policies and practices with regard to the collection and storage of biospecimens/data for future research use

Institution and Respondent(s):
Institution ID #:
Date:

III. USE OF STORED BIOSPECIMENS AND ASSOCIATED DATA FOR GENETIC RESEARCH

Case: For Questions 11 – 15, imagine that a researcher at your institution wants to conduct a new study involving human genetics *using only stored biospecimens/data*.

11. In this situation, what **level of risk** would your IRB typically assign?

When the samples/data the researcher will use are:	No greater than minimal risk	Greater than minimal risk	Unsure/ Don't know
a. Anonymized	○	○	○
b. Coded or de-identified	○	○	○
c. Identified	○	○	○

12. In this situation, would your IRB typically require the researcher to **submit information** about her study so that the IRB can determine, for example, whether the research is exempt or requires IRB review?

When the samples/data the researcher will use are:	Never	Rarely	Sometimes	Usually	Always
a. Anonymized	○	○	○	○	○
b. Coded or de-identified	○	○	○	○	○
c. Identified	○	○	○	○	○

If you answered <u>never</u> to all three—12a and 12b and 12c—skip to Question 16.

Institution and Respondent(s):
Institution ID #:
Date:

13. In this situation, would your IRB typically **review the original consent form** that participants signed (assuming there was one) to determine whether the researcher's proposed new study is within the scope of the potential uses described in the original consent?

When the samples/data the researcher will use are:	Never	Rarely	Sometimes	Usually	Always
a. Anonymized	O	O	O	O	O
b. Coded or de-identified	O	O	O	O	O
c. Identified	O	O	O	O	O

If you answered <u>never</u> to all three—13a and 13b and 13c—skip to Question 16.

14. When your IRB reviews a consent form to determine whether a new proposed study is **within the scope** of the potential uses originally described, what approach does it typically take? Choose one.

_____ Ensure that the proposed study *is consistent* with the uses described (i.e., look for affirmative statements in the original consent form suggesting that the new use would be allowed)

_____ Ensure that the proposed study is *not inconsistent* with the uses described (i.e., ensure there are no statements suggesting that the new use would be contrary to language in the consent)

_____ Unsure / don't know

_____ Other; please specify: ____________________

Institution and Respondent(s):
Institution ID #:
Date:

15. When the IRB determines that a new proposed study is **outside the scope** of the future uses described in the original consent form, what approach would your IRB typically take with regard to the acceptability of each of the following:

	Prohibit this	Discourage this	Permit, but neither encourage/ discourage	Encourage this	Require this
a. *Anonymize* the samples/data (when they were otherwise coded or identified)	O	O	O	O	O
b. *Re-consent* participants for the new use (when the samples/data are coded or identified)	O	O	O	O	O
c. Consider an application for *waiver* of the requirement to obtain informed consent	O	O	O	O	O
d. Other; please specify: _____________________	O	O	O	O	O

e. What is your institution's most preferred approach? _____________ (a – d)

16. Does your IRB have specific procedures that are required to assure **that samples/data are not identifiable** if they are to be coded or anonymized?

_____ Yes
_____ No
_____ Unsure / don't know
_____ Other; please specify: _____________________

17. Please share any thoughts that might help clarify your IRB's policies and practices with regard to the research use of stored biospecimens/data:

Institution and Respondent(s):
Institution ID #:
Date:

IV. SHARING STORED BIOSPECIMENS AND ASSOCIATED DATA FOR GENETIC RESEARCH ACROSS INSTITUTIONS

18. Does your IRB typically require that participants be informed (e.g., in the consent form) that their biospecimens/data may be **shared with other researchers** in the future?

○ Never	○ Rarely	○ Sometimes	○ Usually	○ Always

Case: For Questions 19 – 20, imagine that a researcher at your institution has a collection of biospecimens/data that he would like to *share with a researcher outside your institution*.

19. In situations where your researcher will NOT be involved in the new study (i.e., he is solely providing the biospecimens/data that the outside researcher will study), would your IRB typically require each of the following?

	No/Rarely	Yes/Usually
When the samples/data are **anonymized**:		
a. Submission of information about the new study so *your IRB* can determine, for example, whether the research is exempt or requires IRB review	○	○
b. Submission of documentation of determination/review from *external researcher's IRB*	○	○
When the samples/data are **coded**:		
c. Submission of information about the new study so *your IRB* can determine, for example, whether the research is exempt or requires IRB review	○	○
d. Submission of documentation of determination/review from *external researcher's IRB*	○	○
When the samples/data are **identified**:		
e. Submission of information about the new study so *your IRB* can determine, for example, whether the research is exempt or requires IRB review	○	○
f. Submission of documentation of determination/review from *external researcher's IRB*	○	○

Institution and Respondent(s):
Institution ID #:
Date:

20. In situations where your researcher WILL be involved in the new study (i.e., he will collaborate with the outside researcher on the study), would your IRB typically require each of the following?

	No/Rarely	Yes/Usually
When the samples/data are **anonymized**:		
a. Submission of information about the new study so *your IRB* can determine, for example, whether the research is exempt or requires IRB review	○	○
b. Submission of documentation of determination/review from *external researcher's IRB*	○	○
When the samples/data are **coded**:		
c. Submission of information about the new study so *your IRB* can determine, for example, whether the research is exempt or requires IRB review	○	○
d. Submission of documentation of determination/review from *external researcher's IRB*	○	○
When the samples/data are **identified**:		
e. Submission of information about the new study so *your IRB* can determine, for example, whether the research is exempt or requires IRB review	○	○
f. Submission of documentation of determination/review from *external researcher's IRB*	○	○

Institution and Respondent(s):
Institution ID #:
Date:

Case: For Questions 21 – 23, imagine that a researcher at your institution proposes a study that would involve only biospecimens/data that she would *obtain from a source outside your institution*.

21. Would your IRB typically require each of the following?

	No/Rarely	Yes/Usually
When the samples/data are **anonymized**:		
a. Submission of information about the new study so *your IRB* can determine, for example, whether the research is exempt or requires IRB review?	O	O
b. Details about the *conditions under which the biospecimens/data were originally collected* and stored (e.g., level of IRB oversight, whether and how consent was obtained)	O	O
c. *Review of the original consent form* to determine whether the new study is within the scope of the potential uses described	O	O
d. Evidence that the *outside source's IRB has approved transfer* of samples/data to your institution	O	O
When the samples/data are **coded**:		
e. Submission of information about the new study so *your IRB* can determine, for example, whether the research is exempt or requires IRB review?	O	O
f. Details about the *conditions under which the biospecimens/data were originally collected* and stored (e.g., level of IRB oversight, whether and how consent was obtained)	O	O
g. *Review of the original consent form* to determine whether the new study is within the scope of the potential uses described	O	O
h. Evidence that the *outside source's IRB has approved transfer* of samples/data to your institution	O	O

When the samples/data are **identified**:	No/Rarely	Yes/Usually
i. Submission of information about the new study so *your IRB* can determine, for example, whether the research is exempt or requires IRB review?	O	O
j. Details about the *conditions under which the biospecimens/data were originally collected* and stored (e.g., level of IRB oversight, whether and how consent was obtained)	O	O
k. *Review of the original consent form* to determine whether the new study is within the scope of the potential uses described	O	O
l. Evidence that the *outside source's IRB has approved transfer* of samples/data to your institution	O	O

22. Thinking about the situations in which a researcher at your institution wanted to obtain biospecimens/data from an outside institution, how often has it occurred that the biospecimens/data were collected and/or stored under policies that **differed substantively** with those at your institution? Choose one.

_____ Never
_____ Rarely
_____ Sometimes
_____ Often
_____ Always
_____ I am unsure whether or not this has occurred
_____ We do not require review of materials from outside institutions
_____ Other; please specify: ____________________

If your IRB has <u>never</u> encountered conflicting policies, skip to Question 24.

Institution and Respondent(s):
Institution ID #:
Date:

23. In situations where there is a substantive policy difference between institutions, what approach would your IRB typically take with regard to the acceptability of each of the following:

	Prohibit this	Discourage this	Permit, but neither encourage/ discourage	Encourage this	Require this
a. *Anonymize* the samples/data (when they were otherwise coded or identified)	O	O	O	O	O
b. *Re-consent* participants for the new use (when the samples/data are coded or identified)	O	O	O	O	O
c. Consider an application for *waiver* of the requirement to obtain informed consent	O	O	O	O	O
d. Other; please specify: ______________________	O	O	O	O	O

e. What is your institution's most preferred approach?_____________ (a-d)

24. How much do you agree or disagree with the following statement: *Substantive differences in IRB policies between institutions are a barrier to collaborative genetic research.*

O	O	O	O	O
Strongly disagree	Disagree	Neither agree nor disagree	Agree	Strongly agree

Institution and Respondent(s):
Institution ID #:
Date:

25. Thinking about collaborative genetic research between institutions, how often has your institution entered into an Inter-Institutional Agreement (i.e., an agreement whereby one institution agrees to rely on another institution's IRB review of the research)? Choose one.

_____ Never
_____ Rarely
_____ Sometimes
_____ Often
_____ Always
_____ I am unsure whether or not this has occurred
_____ Other; please specify: ____________________

26. Please share any thoughts that might help clarify your IRB's policies and practices with regard to sharing stored biospecimens/data:

27. In the standard consent-to-treat form in use at your institution's health care facilities, which of the following is most commonly used regarding the storage and potential research use of **residual biospecimens collected for a clinical purpose**? **Choose one**.

_____ Disclosure (no choices) (i.e.,form discloses possibility of research use but does not offer a choice about such use)
_____ Disclosure with opt in (i.e., form discloses possibility but research use cannot occur unless patient opts to allow this)
_____ Disclosure with opt out (i.e., form discloses possibility and research use occurs unless opts to refuse this)
_____ No disclosure (i.e., form does not disclose possibility and research use can still occur)
_____ Researchers must get specific consent (i.e., research use of residual biospecimens must be addressed separately from consent-to-treat form)
_____ Unsure / don't know
_____ Other; please specify: ____________________

Institution and Respondent(s):
Institution ID #:
Date:

28. With regard to the need for re-consent for continued storage and research use of coded or identifiable biospecimens/data collected from **children** when they reach adulthood, what approach does your IRB typically take? (assume identifying information is available)

○	○	○	○	○
Prohibit this	Discourage this	Permit, but neither encourage/discourage	Encourage this	Require this

29. If a research participant who contributed biospecimens/data to a stored collection wants to **withdraw consent**, what approach would your IRB typically take with regard to the acceptability of each of the following:

	Prohibit this	Discourage this	Permit, but neither encourage/ discourage	Encourage this	Require this
*For **biospecimens** and associated clinical data:*					
a. Destruction/permanent removal from collection	○	○	○	○	○
b. Anonymization and continued use	○	○	○	○	○
c. Other; please specify: ____________________	○	○	○	○	○

What is your institution's preferred approach using the choices a – c ? ______________

Q29, continued

	Prohibit this	Discourage this	Permit, but neither encourage/ discourage	Encourage this	Require this
*For **data** derived from analysis of biospecimens prior to the withdrawal*					
d. Destruction/permanent removal of all data from the individual in the dataset from collection	O	O	O	O	O
e. Anonymization of data and continued use	O	O	O	O	O
f. Continued use of coded/identifiable data acquired before withdrawal	O	O	O	O	O
g. Other; please specify: ____________________	O	O	O	O	O

What is your institution's preferred approach using the choices d – g ___________

30. When biospecimens/data have been shared with a researcher outside your institution, who has the responsibility to ensure the appropriate action is taken when a research participant wants to **withdraw consent**?

_____ *Your* institution / IRB / researcher
_____ The *outside* institution / IRB / researcher
_____ Your institution and the outside institution
_____ Other; please specify: _____________________

Institution and Respondent(s):
Institution ID #:
Date:

31. Does your IRB have specific policies or usual practices in place concerning the **disclosure of individual research results** (e.g., from the analysis of biospecimens/data) to research participants?

_____ Yes
_____ No
_____ Unsure / don't know
_____ Other; please specify: ____________________

32. When biospecimens/data have been shared with a researcher outside your institution, who has the responsibility to ensure the appropriate action is taken with regard to the **disclosure of individual research results**?

_____ *Your* institution / IRB / researcher
_____ The *outside* institution / IRB / researcher
_____ Your institution and the outside institution
_____ Other; please specify: ____________________

Institution and Respondent(s):
Institution ID #:
Date:

33. How important is each of the following to the **development of your IRB's policies and practices** concerning the collection, storage, and research use of human biospecimens and data?

	Not important	Of little importance	Somewhat important	Important	Very important
a. OHRP policies/guidance documents	○	○	○	○	○
b. Institutional experts in this domain	○	○	○	○	○
c. Empirical data concerning the effects of different policy options	○	○	○	○	○
d. Consensus statements from ad hoc expert groups	○	○	○	○	○
e. Consensus statements from professional organizations	○	○	○	○	○
f. Advice and/or requirements of institutional legal counsel	○	○	○	○	○
g. AAHRPP accreditation standards	○	○	○	○	○
h. Other; please specify: ______________________	○	○	○	○	○

34. Please rank the top 3 influences from among options a – h in Question 33 (you can refer to each of the options using its corresponding letter):
#1 ____________
#2 ____________
#3____________

Institution and Respondent(s):
Institution ID #:
Date:

35. **Does your IRB have written policies or guidance concerning the: 1) collection, 2) storage, 3) use, and/or 4) sharing of biospecimens/data in genetic research and/or more general policies that apply to genetic research?**

_____Yes
_____No
_____Unsure / don't know

***If yes*, we would very much appreciate your willingness to share copies of all relevant policy documents/guidance with us. There are several options for doing so, including**:

- Email your policies as an attachment to the Principal Investigator, Leona Cuttler, M.D. at leona.cuttler@case.edu
- If your policies are posted on a publicly accessible web page, please provide us with the URL
- Fax a copy of each policy document to Leona Cuttler at 216-844-8900

Your documents will be kept confidential and your institution will not be identified in any reports of the results of this project.

36. As part of this project, we will be conducting **follow-up phone interviews** with IRB administrative directors concerning their IRB's policies and practices with regard to banking and sharing biospecimens/data. If you would prefer that we *not* contact you to invite your participation in an interview, please check the box below.

_____ I prefer NOT to be contacted to discuss participating in an interview

37. **Is your IRB** AAHRPP-accredited?

_____ Yes
_____ No

Institution and Respondent(s):
Institution ID #:
Date:

SURVEY IS NOW COMPLETE

Thank you for taking the time to share your experiences and opinions concerning the protection of research participants in collaborative genetic research involving the collection, storage, and use of biospecimens and associated data.

Your responses will be invaluable for helping us identify policy priorities that could strengthen and facilitate collaborative genetic research across academic institutions in a way that optimally protects human subjects.

Your responses will be kept confidential and results will be reported in aggregate only. If you would like to receive the aggregate results of the study, please check the box and indicate the preferred address for mailing.

I would like to receive the aggregate survey results. If yes, please

- check the box ☐ **and**
- **include your e-mail address**: __________________
